# CircRNA Expression Profiles and the Potential Role of CircZFP644 in Mice With Severe Acute Pancreatitis via Sponging miR-21-3p

**DOI:** 10.3389/fgene.2020.00206

**Published:** 2020-03-12

**Authors:** Yi Yang, Jiandong Ren, Qilin Huang, Jun Wu, Xiaohui Yuan, Wen Jiang, Yi Wen, Lijun Tang, Hongyu Sun

**Affiliations:** ^1^Department of General Surgery & Pancreatic Injury and Repair Key Laboratory of Sichuan Province, The General Hospital of Western Theater Command (Chengdu Military General Hospital), Chengdu, China; ^2^College of Medicine, Southwest Jiaotong University, Chengdu, China; ^3^School of Medicine, University of Electronic Science and Technology of China, Chengdu, China

**Keywords:** circRNA, severe acute pancreatitis, RNA-seq, CircZFP644, ceRNA, miRNA, miR-21

## Abstract

Severe acute pancreatitis (SAP) is the most serious type of pancreatitis with high morbidity and mortality. The underlying mechanism behind SAP pathogenesis is complex and remains elusive. Circular RNAs (circRNAs) are emerging as vital regulators of gene expression in various diseases by sponging microRNAs (miRNAs). However, the roles of circRNAs in the pathophysiology of SAP remain unknown. In the present study, next-generation RNA sequencing was utilized to identify circRNA transcripts in the pancreatic tissues from three SAP mice and three matched normal tissues. The differentially expressed circRNAs were confirmed by real-time PCR, and the biological functions of their interaction with miRNAs and mRNAs were analyzed. Our results demonstrate that 56 circRNAs were differentially expressed in SAP mice compared with normal controls. Six differentially expressed circRNAs were confirmed with the sequencing data. Importantly, we characterized a significantly downregulated circRNA derived from the ZFP664 gene in SAP. CircZFP644 was found to be negatively correlated with miR-21-3p, with a perfectly matched binding sequence to miR-21-3p. In conclusion, CircZFP644 may play an important role in the pathogenesis of SAP through sponging miR-21-3p. Our findings may provide novel insights regarding the workings of the pathophysiological mechanism of SAP and offer novel targets for SAP.

## Introduction

Acute pancreatitis (AP) is a common inflammatory disorder of the pancreas with high morbidity and mortality (Peery et al., 2019). Approximately 20% of patients with AP develop severe acute pancreatitis (SAP) characterized by extensive pancreatic necrosis and multiple organ failure, resulting in mortality rates as high as 30% (Forsmark et al., 2016). Unfortunately, the pathophysiology of SAP is complex and remains elusive. Therefore, it is critical to address the biological processes and molecular mechanisms underlying the development and progression of SAP so that novel preventive or therapeutic strategies can be created.

Non-coding RNAs (ncRNAs), such as microRNAs (miRNAs) and circular RNAs (circRNAs), have emerged as vital regulators of gene expression under multiple physiological and pathological conditions (Beermann et al., 2016). Among them, miRNAs are the most widely characterized and studied. A body of evidence indicates that altered expression of miRNAs is implicated in the pathogenesis of AP in animal models and human specimens (Xiang et al., 2017; Yang et al., 2019). Furthermore, functional studies also confirm that knockdown of certain miRNAs (e.g., miR-21) can be conducive to the reduction of damage caused by SAP (Ma et al., 2015; Li et al., 2018a). Although the roles of miRNAs have been well characterized, the precise mechanisms of miRNAs in SAP remain largely unexplored.

There has recently been great interest in circRNAs, which are a novel class of ncRNAs. CircRNAs are characterized by forming covalently closed loop structures through backsplicing or exon-skipping events (Chen and Zhao, 2018). They possess stability that is higher than that of linear RNA, as well as tissue or developmental-stage specific expression patterns. To date, numerous circRNAs have been identified in different tissues and diseases by RNA sequencing (RNA-seq), including normal pancreas tissue and in diabetes mellitus (Xu et al., 2017; Stoll et al., 2018). Notably, circRNAs can function as miRNA sponges to regulate the expression of miRNA and its target genes through the competing endogenous RNA (ceRNA) network (Han et al., 2018). Increasing evidence has revealed that circRNA-associated ceRNA networks play important roles in a variety of diseases, such as cancer, heart disease, and diabetes mellitus (Zhang et al., 2018; Zhong et al., 2018). Apart from acting as miRNA sponges, circRNAs can also influence transcription and splicing, bind to RNA-binding proteins and even be translated (Li et al., 2018b). However, the potential circRNAs involved in SAP are still unknown.

In this study, first, we investigated the expression profiling of circRNA in necrotic pancreas tissues from SAP model mice and matched normal tissues by RNA-seq. Then, using quantitative reverse transcription-polymerase chain reaction (qRT-PCR), we further verified the significantly differentially expressed circRNAs in SAP. Interaction network between the validated circRNAs and miRNAs revealed that decreased CircZFP644 may play an important role in development of SAP by upregulating miR-21-3p. Subsequent results showed that CircZFP644 remarkably decreased while miR-21-3p significantly increased in the pancreatic tissues from SAP mice and three matched binding sequence was found between CircZFP644 and miR-21-3p.

## Materials and Methods

### Animals

All experiments were performed according to protocols approved by the Institutional Animal Care and Use Committee at the General Hospital of Western Theater Command. Male C57BL/6 mice at 7–8 weeks were purchased from Dashuo Animal Science and Technology Co., Ltd. (Chengdu, China). They were maintained using the individually ventilated cage (IVC) system and fed with standard laboratory food and water *ad libitum* for 3 days before the experiments. Animals were fasted overnight but had free access to water before the modeling.

### Preparation of SAP Model

The method of model preparation is similar to that we previously reported (Liu et al., 2018). Briefly, mice were randomly divided into control group and SAP group and anesthetized with 5% isoflurane (via induction box) prior to surgery. The SAP model was induced by a standardized pressure-controlled retrograde infusion of 4% sodium taurocholate into the biliopancreatic duct by using a micro infusion pump. All animals were sacrificed and sampled 24 h after induction of pancreatitis.

### Pancreatic Histological Analysis

Tissues of the pancreatic head were fixed in 4% formaldehyde phosphate overnight and then dehydrated and paraffin embedded. For hematoxylin and eosin staining, 4 μm sections were stained with the dye and then examined by light microscopy, and the histopathological score was evaluated by using a previously described scoring system (Schmidt et al., 1992). The scores were averaged for five individual slides from each pancreas.

### Amylase and Lipase Measurement

The levels of serum amylase and lipase were quantified from blood collected from the tail vein using commercial kits (Nanjing Jiancheng Bioengineering Institute, China) according to the manufacturer’s protocols.

### Samples and RNA Isolation

The control and SAP groups each contained three mice. RNA was isolated from homogenized pancreatic tissue 24 h after surgery using TRIzol reagent (Ambion) according to the manufacturer’s instructions. The concentration of RNA was measured at OD260/280 using a NanoDrop ND-2000 spectrophotometer (Thermo, Waltham, MA, United States). The integrity of the RNA was assessed using denaturing agarose gel electrophoresis.

### RNA Library Construction and CircRNA Sequencing

The total RNA of3 mice from SAP group and 3 mice from control group was used to prepare the circRNA sequencing library, according to the following steps: (1) 5 μg total RNA was pretreated to enrich circRNAs using the CircRNA Enrichment Kit (Cloud-Seq Inc., Shanghai, China). (2) RNA libraries were constructed from the treated RNAs using the TruSeq Stranded Total RNA Library Prep Kit (Illumina, San Diego, CA, United States) according to the manufacturer’s instructions. (3) Quality control and quantification of libraries were performed by the BioAnalyzer 2100 system (Agilent Technologies, Inc., Santa Clara, CA, United States). (4) The denatured libraries as single-stranded DNA molecules were captured on Illumina flow cells, amplified *in situ* as clusters, and finally sequenced for 150 cycles on an Illumina HiSeq 4000 Sequencer according to the manufacturer’s instructions.

### CircRNA Sequencing Analysis

Paired-end reads were harvested by the Illumina HiSeq 4000 sequencer and quality controlled using Q30 (Ewing et al., 1998). After 3′ adaptor trimming and removal of low quality reads using Cutadapt software (Kechin et al., 2017), the high quality trimmed reads were aligned with the reference genome/transcriptome using BWA−MEM software (Cloud-Seq, Inc., Shanghai, China) and circRNAs were detected and annotated with CIRI software (Cloud-Seq, Inc., Shanghai, China) (Gao et al., 2015). Raw junction reads for all samples were normalized to the number of total mapped reads and log2 transformed. CircRNAs exhibiting fold changes more than 2.0 with *P*-values less than 0.05 were classified as significantly differentially expressed circRNAs.

### qRT-PCR Analysis

Pancreatic tissues from 6 SAP mice and 6 healthy mice were used for verification. qRT-PCR with SYBR Green (Takara, Dalian, China) analysis was used to validate the expression of miR-21-3p and the differentially expressed circRNAs. These circRNAs were chosen based on their functional roles and respective fold-change and *p*-values, including CircAMY2a5 (chr3:113256960-113258035-), CircZFP644 (chr5:106634831- 106638635-, mmu_circ_0000663), CircDTNB (chr12:3617606-3648389+, mmu_circ_0000349), CircCELA3b (chr4:137404385-137423389-), CircARHGEF38 (chr3:133137266-133149650-), CircSRPK2 (chr5:23540375- 23565265-, mmu_circ_0001325), and the primers are listed in Table 1. For RNase R treatment, 2 μg total RNA was treated with or without 20 U/μL RNase R (Epicentre, Shanghai, China) for 20 min at 37°C. For qRT-PCR analysis, total RNA was reverse-transcribed into complementary DNA (cDNA) using the Invitrogen Superscript cDNA Synthesis kit (Invitrogen, Carlsbad, CA, United States). Reactions were performed according to the manufacturer’s instructions. The expression of gene GAPDH was used as a reference for circRNA normalization. The relative expression level of the miR-21-3p was analyzed by the same real-time qRT-PCR method used for validation of the circRNAs. U6 was used as an endogenous control gene for miR-21-3p. The relative expression of circRNA and miRNA was calculated according to the 2^–Δ^ ^Δ^ ^Ct^ method.

**TABLE 1:** Sequences of primers used for quantitative real-time polymerase chain reaction analysis of circRNA levels.

**Name**	**Primer type**	**Primer sequence**
CircAMY2a5	Forward	TGTTTTGGAGATTTGCTGTGAGA
	Reverse	GTCCTCACTTACCTAACAAAGAAAA
CircZFP644	Forward	CCGTTGATCTATCAGCCACA
	Reverse	GCACCAGTAATGTCGGTGTTT
CircDTNB	Forward	GCTCTGCCAGAACTGCTTTT
	Reverse	CCATTGGAATCTGACATCTGG
CircCELA3b	Forward	CCCCAGCAATAACATCGC
	Reverse	CAGACTGGATATCGCCGC
CircARHGEF38	Forward	CCTCCTCCGGGACTTGAT
	Reverse	CCCATGTCCAGCAGGTTC
CircSRPK2	Forward	TCAAGGCCTCCCAGTACG
	Reverse	GTGGTGGTGGTGGAGGAG
GAPDH	Forward	AAGGTCATCCCAGAGCTGAA
	Reverse	CTGCTTCACCACCTTCTTGA
miR-21-3p	Forward	GAAATGC-CTCACAGCTATCGT
	Reverse	CCTCCACAAAGAGCCACC
U6	Forward	CGCTTCGGCAGCACATATAC
	Reverse	AAATATGGAACGCTTCACGA

### GO and KEGG Pathway Analysis

The host genes of differentially expressed circRNAs were selected to analyze their potential biological roles through gene ontology (GO) functional annotation and Kyoto Encyclopedia of Genes and Genomes (KEGG) pathway analysis (Kanehisa et al., 2017). The GO program has developed a structured and controlled vocabulary for community annotation genes, gene products, and sequences (Ashburner et al., 2000). It contains three parts: biological process (BP), molecular function (MF), and cell component (CC). Fisher’s exact test was applied to analyze the probabilities with clusterProfiler packages in R/bioconductor. The selection criteria include fold change ≥ 2.0 and *p* < 0.05.

### CircRNA-miRNA Networks Analysis

CircRNAs containing miRNA binding sites and the corresponding miRNAs were subjected to analysis with Cytoscape software (Cloud-Seq, Inc., Shanghai, China) to construct circRNA-miRNA networks and demonstrate interactions. MiRNA binding sites on circRNAs and the putative target genes of these miRNAs were all predicted by custom-written software based on Targetscan and Miranda (Betel et al., 2008) software (Cloud-Seq Biotech Ltd., Co., Shanghai, China).

### Sanger Sequencing

To obtain more reliable PCR results, the PCR amplification products were separated by electrophoresis in agarose gels. Briefly, the samples were loaded into precast wells in the gels and then subjected to electrophoresis. Subsequently, the PCR products were stained with ethidium bromide and observed under ultraviolet light. Finally, the gels were dissected and the PCR products were recovered for subsequent sequencing experiments. For Sanger sequencing, the specific PCR products were extracted and directly purified with the Mag-MK Gel DNA Purification Kit (Sangon Biotech, Shanghai, China) according to manufacturer’s instructions. The sequences of the products were determined by an ABI 3730 DNA sequencer (Applied Biosystems, CA, United States).

### Statistical Analysis

Statistical analyses were performed with the GraphPad Prism 7 (GraphPad, CA, United States) and SPSS 22.0 software packages (SPSS, IL, United States). Statistically significant differences between groups were estimated by Student’s *t*-test. Fisher’s exact test was applied in the GO and KEGG pathway analyses. The results were evaluated using Spearman’s correlation coefficient test. All values are expressed as the mean ± standard error of the mean; *p* < 0.05 was considered statistically significant.

## Results

### Evaluation of the SAP Model

**FIGURE 1:**
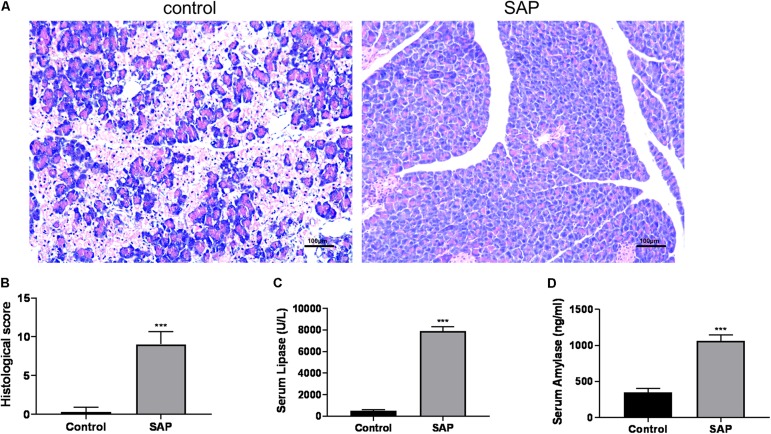
Evaluation of the SAP model. **(A)** Representative histological sections of mouse pancreas stained with hematoxylin from control (left) and SAP (right) groups (100x). **(B)** The histological score of pancreatic tissue. **(C,D)** Levels of serum lipase and amylase, respectively. ***p < 0.001, vs. control group, *n* = 6 per group.

The featured histopathological changes were found in the pancreatic tissues from the SAP group, including pancreatic lobule interstitial edema, granulocyte infiltration, and extensive acinar cell necrosis (Figure 1A), and the corresponding histopathological scores are also presented (Figure 1B). By contrast, when viewed under a light microscope, pancreases from the control group were intact with a normal structure. The levels of serum lipase and serum amylase in the SAP group were significantly higher than those in the control group (*P* < 0.05) (Figures 1C,D). These results suggested that a reliable SAP mouse model was established.

### Overview of CircRNA Expression Profiles in SAP

**FIGURE 2:**
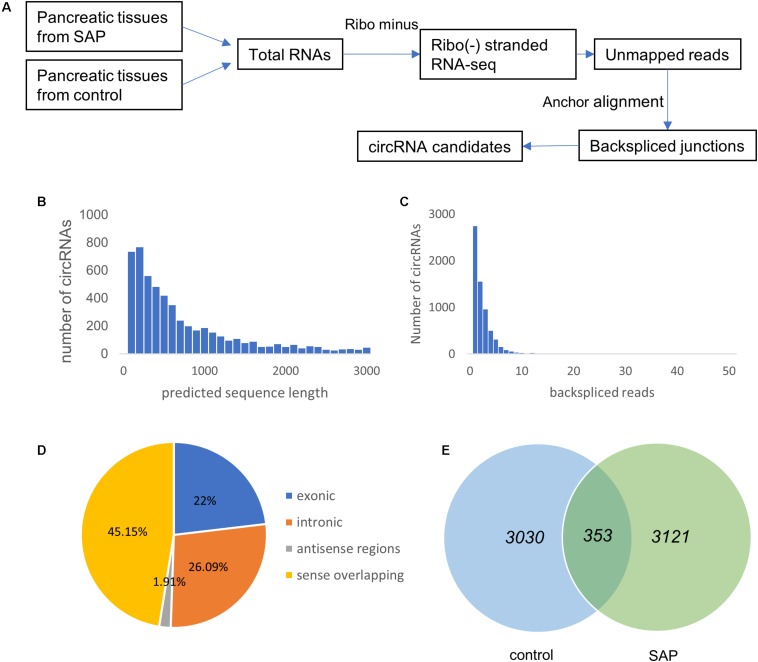
Overview of circRNA expression profiles in SAP. **(A)** The experimental procedures for obtaining circRNA candidates. **(B)** The number of circRNAs (*y*-axis) based on the backspliced reads (*x*-axis). **(C)** The number of circRNAs (*y*-axis) based on the predicted length of the backspliced reads (*x*-axis). **(D)** The percentage of differentially expressed circRNAs based on their categories of circular components. **(E)** Venn diagram showing the number of common and specific circRNAs between the SAP and control groups.

RNA-seq analysis was utilized to identify circRNA transcripts in the pancreatic tissues from three SAP mice and three healthy mice (Figure 2A). A total of 6504 distinct circRNA candidates were found in these tissues, of which 3763 circRNAs contained at least two unique back-spliced reads (Figure 2B). Next, we analyzed the characteristics of these circRNAs. Most of the circRNA candidates were less than 1500 nucleotides (nts) in length (Figure 2C). As shown in Figure 2D, these circRNAs were located at various genomic regions, including exonic (22%), intronic (26.09%), sense overlapping (45.15%), and antisense (1.91%) regions. There were 3121 circRNAs specifically expressed in the SAP group, 3030 circRNAs specifically expressed in the control group, and 353 circRNAs commonly expressed in both groups (Figure 2E).

### Differentially Expressed CircRNAs in SAP

**FIGURE 3:**
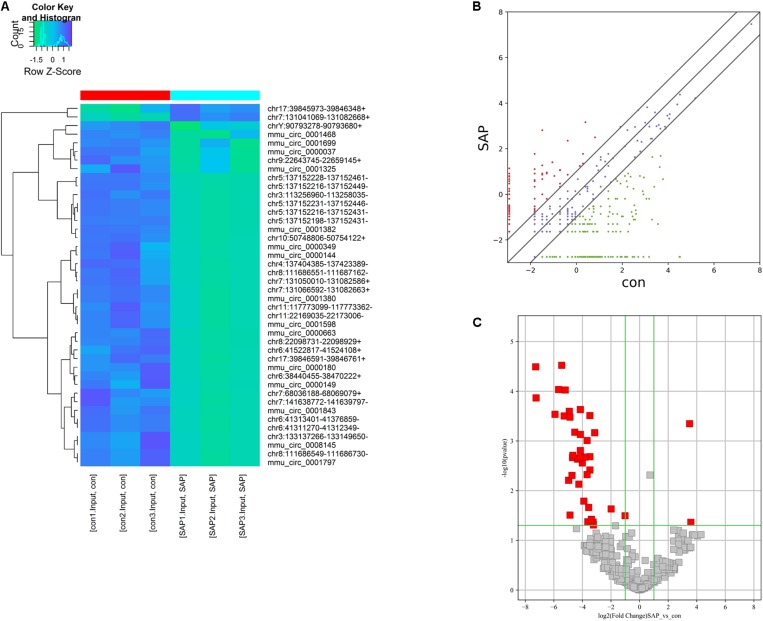
Characterization of circRNA expression profiles of SAP mice and healthy mice. **(A)** Hierarchical clustering graph showing the expression patterns of circRNAs in pancreatitic tissues of SAP and the control. **(B,C)** Scatter and volcano plot show the significant differentially expressed circRNAs between the SAP and the control groups and identify fold changes greater than 2.0 and *p*-values less than 0.05.

On the basis of the circRNA expression analysis, 56 circRNAs (*p* < 0.05, fold change ≥ 2) were found to be significantly differentially expressed in the SAP group compared with the control group. Among these differentially expressed circRNAs, 2 circRNAs were upregulated and 54 circRNAs were downregulated in the SAP group compared to healthy controls. Of these, the fold-changes of 42 circRNAs were at least greater than 10 (Supplementary Table S1). As seen in Figure 3A, the expression patterns of circRNAs in the pancreatic tissues of SAP mice and control mice were classified by unsupervised hierarchical clustering. The scatter plot demonstrates the variation in the circRNA expression level between the two groups (Figure 3B). A volcano plot shows that the significantly differentially expressed circRNAs between the two groups were identified with fold-changes greater than 2.0 and *p-*values less than 0.05 (Figure 3C).

### Function of the Differentially Expressed CircRNAs in SAP

**FIGURE 4:**
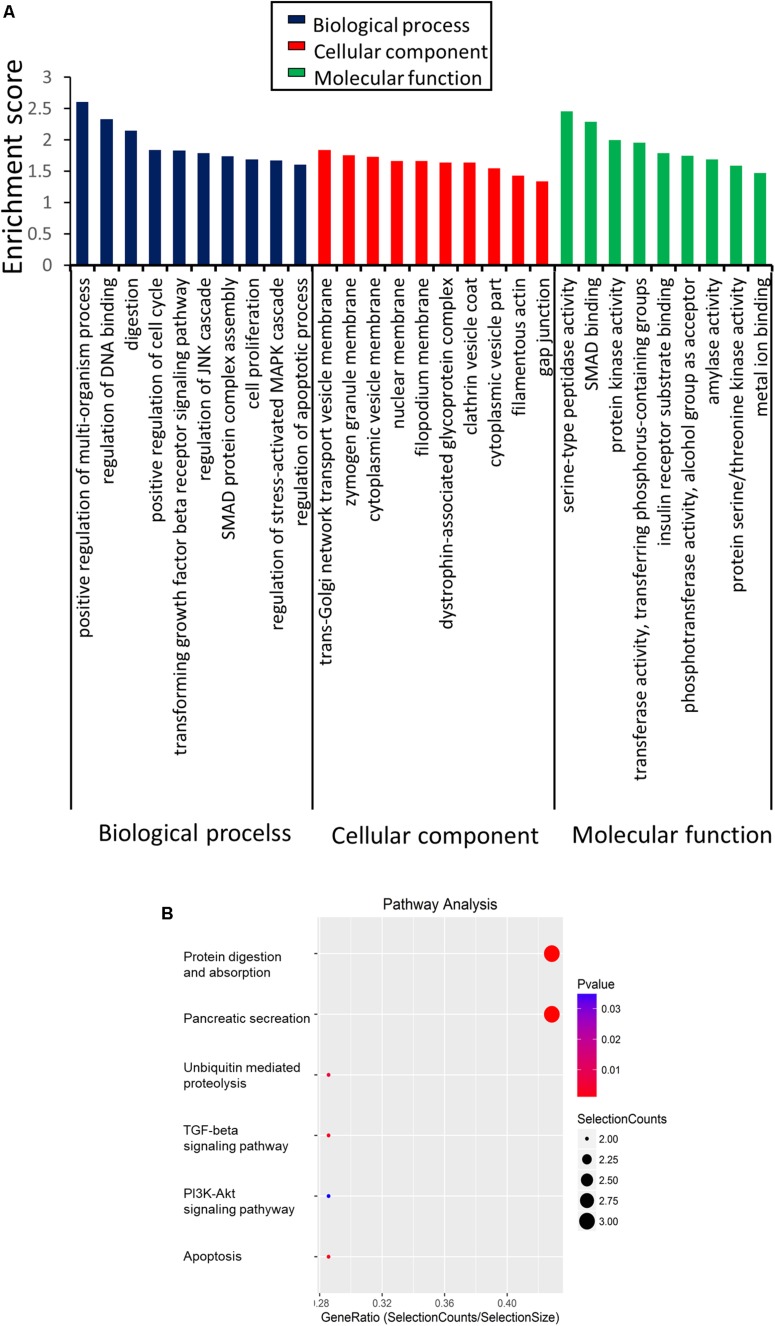
Functions of the differentially expressed circRNAs in SAP by GO and KEGG analysis. **(A)** GO analysis of genes related to differentially expressed circRNAs. GO annotations of the linear counterparts of upregulated and downregulated circRNAs in the cellular component, molecular function, and biological process terms. **(B)** KEGG pathway analysis of the differentially expressed circRNAs.

Given that the overwhelming majority of dysregulated circRNAs were downregulated in the SAP mice compared with the normal group, we mainly focused on the 54 significantly downregulated circRNAs. To investigate the potential function of the differentially regulated circRNAs, we first performed GO enrichment analysis, including biological processes (BP), cellular components (CC), and molecular functions (MF) (Figure 4A). For BP, the major enriched and meaningful GO terms were multi-organism process, DNA binding, and digestion. In terms of CC, trans-Golgi network transport vesicle membrane, zymogen granule membrane, and cytoplasmic vesicle membrane were the top three terms. For MF analysis, serine-type peptidase activity, SMAD binding, and protein kinase activity were the most enriched molecular functions.

Then, KEGG analysis was conducted for defining the pathways relevant to the dysregulated circRNAs in the pathological process of SAP. As shown in Figure 4B, protein digestion and absorption (3 genes), apoptosis (3 genes), TGF-beta signaling (3 genes), and the PI3K-Akt signaling pathway (2 genes) were the major signaling pathways associated with the dysregulated circRNAs.

### Validation of Differentially Expressed CircRNAs in SAP

**FIGURE 5:**
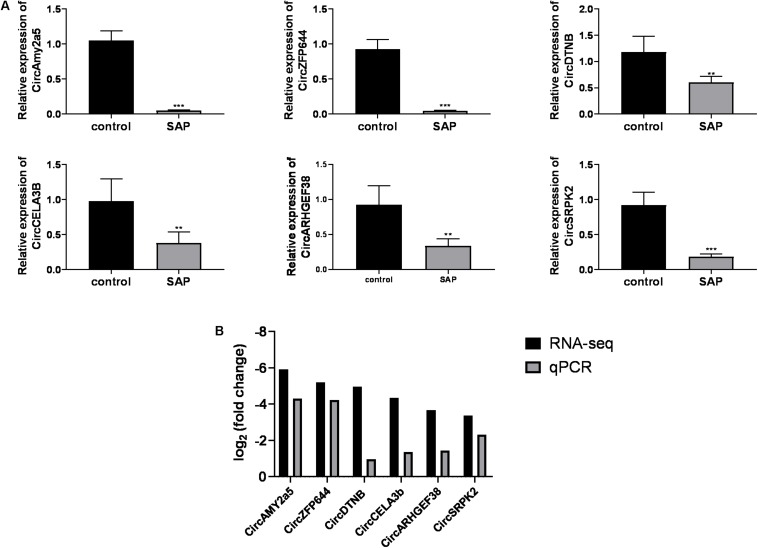
Validation of differentially expressed circRNAs. **(A)** The relative expression levels of selected circRNAs detected by the qRT-PCR method. GAPDH was used as a housekeeping gene for normalizing changes in specific gene expression. **(B)** The comparison between the RNA-Seq data and quantitative real-time polymerase chain reaction results. ***p < 0.001, **p < 0.01, vs. control group. *n* = 6 per group. qRT-PCR, Quantitative reverse transcription-polymerase chain reaction.

Considering that 54 out of 56 differentially expressed circRNAs were downregulated in SAP mice, we mainly focused on the downregulated circRNAs. Based on the ranking order of the multiple integrated factors described above, six downregulated circRNAs were selected for validation by qRT-PCR. The expression levels of CircAMY2a5, CircZFP644, CircDTNB, CircCELA3b, CircARHGEF38, and CircSRPK2, were significantly downregulated in the SAP group compared with the normal control (Figure 5A). Taken together, the qRT-PCR results for these six circRNAs were consistent with the RNA-seq data (Figure 5B).

### The Interaction Network Between CircRNA and miRNA in SAP

**FIGURE 6:**
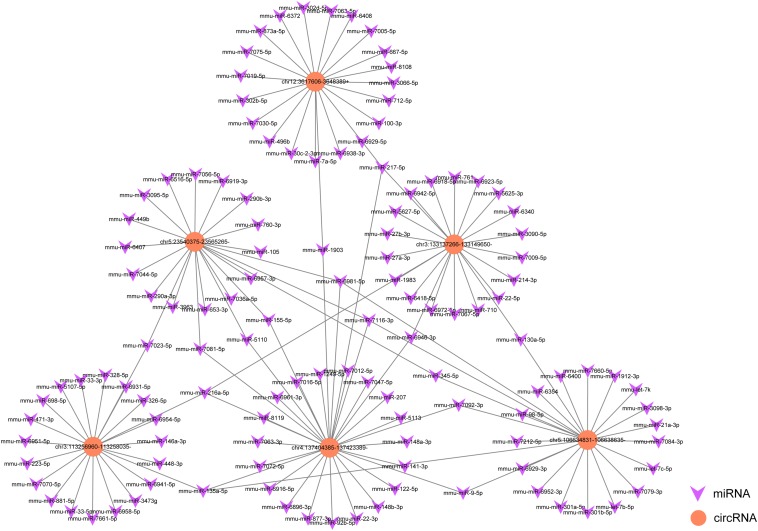
A map showing the interaction network contained 6 validated circRNAs and their around 20 target miRNAs with the most stable binding in SAP. Circles represent circRNAs, arrowheads represent miRNAs.

To explore the underlying mechanism of circRNAs in mediating mRNA based on miRNA in SAP, an interaction network between circRNAs and miRNAs was constructed using TargetScan and miRanda analysis. Six of the validated circRNAs were included in this ceRNA analysis. In the network map, the miRNAs that potentially bind to the 6 circRNAs are clearly shown in Figure 6. The data may provide a novel perspective for us to study the specific mechanism of circRNAs in the pathological process of SAP.

### Characterization of CircZFP644

**FIGURE 7:**
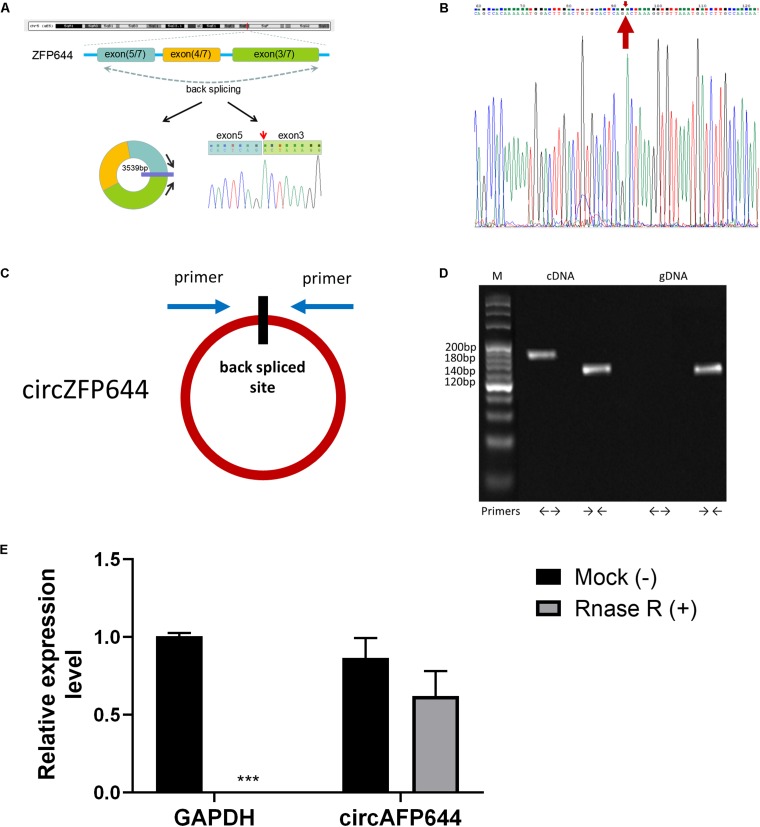
Characterization and expression analysis of CircZFP644. **(A)** Schematic diagram of CircZFP644 formed by back-splicing from the mouse ZFP644 gene at chromosome 5. **(B)** Schematic view illustrating the design of primers for CircZFP644 used in qRT-PCR. **(C)** The junction sequences of CircZFP644 were validated by Sanger sequencing. **(D)** Divergent primers detected CircZFP644 in complementary DNA (cDNA), but not in genomic DNA (gDNA). **(E)** RNA from mouse cardiac tissue was incubated with RNase R or buffer only (Mock). After digestion, the RNAs were purified. The levels of CircZFP644 and GAPDH mRNA analyzed by qRT-PCR after incubation with RNase R or buffer only (Mock). ***p < 0.001, vs. control group. *n* = 6 per group.

Based on the above-described results, we focused on a significantly dysregulated circRNA, circRNA.0001382, which was 36-fold downregulated in SAP. We found that mmu_circ_0001382 was spliced from the ZFP644 gene on chromosome 5:106634831-106638635(-), and its total length is 3539 nt (Figure 7A). We thus termed this circRNA as “CircZFP644.” The junction regions of CircZFP644 were amplified using specific qRT-PCR primers (Figure 7B), and Sanger sequencing of the PCR products confirmed its back-spliced junction (Figure 7C). Using specific divergent primers for CircZFP644, a specifically amplified PCR product was seen from both cDNA and gDNA, while it was only amplified from cDNA through divergent primers (Figure 7D). Resistance to digestion by RNase R further confirmed that CircZFP644 existed in a circular form (Figure 7E). Taken together, these results indicate that CircZFP644 is a stable circRNA derived from the ZFP644 gene locus.

### Interaction Between CircZFP644 and miR-21-3p

**FIGURE 8:**
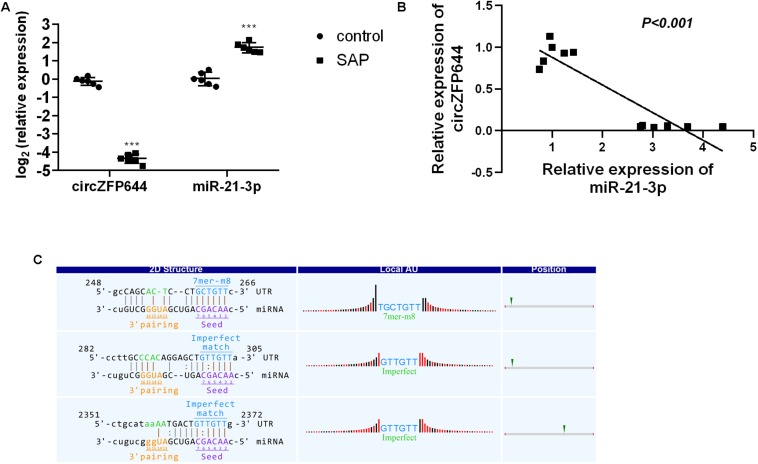
Interaction between pancreatic CircZFP644 and miR-21-3p in SAP. **(A)** The relative expression levels of CircZFP644 and miR-21-3p between control and SAP groups by qRT-PCR. The data are expressed as the mean ± SD. **(B)** The tendency for negative correlation between CircZFP644 and miR-21-3p. **(C)** The interaction between CircZFP644 and miR-21-3p was predicted by TargetScan and miRanda. The “2D Structure” column shows the binding sequence of CircZFP644 and miR-21-3p as a perfect match seed type (7-mer-m8) with two imperfect match sites. The “Local AU” column displays 30 nucleotides in the upstream and downstream of the seed sequence. The “Position” column indicates the probable position of the miR-21-3p response element on the linear presentation of CircZFP644.

Given that miR-21-3p is the most studied miRNA in the pathogenesis of AP and reported to be significantly increased in SAP mice and involved in the necrotic process of SAP (Wang et al., 2018), we thus investigated the correlation between CircZFP644 and miR-21-3p. As seen in Figure 7A, the expression of miR-21-3p was remarkably higher in SAP pancreatic tissues as compared to normal tissues, and inversely, CircZFP644 displayed a significant decrease in SAP than that in the normal control. Notably, negative correlation was found between CircZFP644 and miR-21-3p (Figure 8B). Moreover, the binding sequence between CircZFP644 and miR-21-3p was analyzed by Target Scan and miRanda, and it was determined that there were 3 binding sites found between CircZFP644 and miR-21-3p (Figure 8C).

**FIGURE 9:**
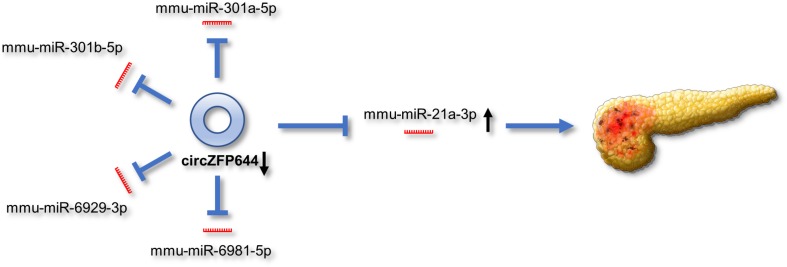
Schematic view of the potential role of CircZFP644 in the pathogenesis of SAP. CircZFP644 could function as miRNA sponges to regulate multiple miRNAs such as miR-301a-5p, miR-301b-5p, miR-6929-3p, miR-6981-5p, and miR-21-3p. Notably, CircZFP644 could sponge miR-21-3p and modulate its target genes, ultimately resulting in the necrotic pancreatitis.

Based on the above findings, we propose a schematic model for the potential role of CircZFP644 in the pathogenesis of SAP (Figure 9). CircZFP644 could regulate the expression of multiple miRNAs such as miR-301a-5p, miR-301b-5p, miR-6929-3p, miR-6981-5p, and miR-21-3p, and involve itself in the necrotic process of SAP. Notably, CircZFP644 could sponge miR-21-3p and modulate its target genes, ultimately resulting in necrotic pancreatitis.

## Discussion

To date, no effective therapeutic options are available for the clinical treatment of SAP. Therefore, it is crucial to investigate the underlying mechanisms of SAP for the development of effective therapeutics. In the present study, we identified 56 circRNAs with significantly differential expression and confirmed that six circRNAs exhibited significant downregulation in the pancreatic tissues of SAP mice. Importantly, we found a tendency of negative correlation and a perfectly matched binding sequence between CircZFP644 and miR-21-3p, suggesting that CircZFP644 may play a significant role in the pathogenesis of SAP by sponging miR-21-3p. These findings may offer a novel insight into new therapeutic targets for SAP.

Numerous studies have explored the molecular mechanisms underlying SAP, and accumulating evidence indicates that non-coding RNAs (ncRNAs), especially miRNAs, significantly regulate the function of pancreatic acinar cells to exert an influence on the process of SAP. For example, the expression of miR-216a in pancreatic tissue is relatively specific in that its concentration is significantly higher in the pancreas than in other tissues (Kong et al., 2010). Elevated miR-216a during AP activates the PI3K/AKT signaling pathway and aggravates pancreatic tissue damage and systemic inflammation via inhibiting the expression of target genes phosphatase tensin homolog (PTEN) and mothers against decapentaplegic homolog 7 (Smad7) (Zhang et al., 2015). Overexpression of miR-352 causes dysfunction of the autophagic lysosomes in acinar cells and reduces the scavenging efficiency of trypsinogen and trypsin, which leads to premature of trypsinogen and occurrence of AP (Song et al., 2018). Interestingly, when miR-141 was transfected into AP mice, the hyperactive autophagy in acinar cells was suppressed, and the damage in pancreatic tissue was ameliorated (Zhu et al., 2016). Taken together, these findings show the important role of miRNA in SAP pathogenesis and the potential for treatment of SAP by targeting miRNAs. However, the potential function of circRNAs, a novel type of non-coding RNA, in the pathophysiological process of SAP has not been reported.

CircRNAs are widely considered to act as vital regulators under multiple physiological and pathological conditions. Studies have shown that circRNAs are abundantly expressed in pancreatic tissues and are related to the development and progression of pancreatic diseases (Xu et al., 2015; Zhang et al., 2019). Stoll et al. (2018) reported that circHIPK3 and ciRS-7/CDR1as impaired insulin secretion by regulating the expression of key β-cell genes in diabetic conditions. Despite the advancements, the role of circRNAs in SAP pathogenesis remains unclear. In this study, we identified 56 differentially expressed circRNAs in the necrotic pancreatic tissue of SAP mice compared with the normal control tissue, including 2 upregulated circRNAs and 54 downregulated circRNAs. Using qRT-PCR, we confirmed six downregulated circRNAs, which were consistent with the RNA-seq results. To the best of our knowledge, this is the first report that has described the circRNA expression profiles in SAP. These dysregulated circRNAs from our study may provide novel insights regarding the role of circRNAs in the development and progression of SAP.

GO and KEGG analyses were constructed to define the function of the dysregulated circRNAs in SAP pathogenesis. According to our results, we found that these dysregulated circRNAs were enriched in several pathways, including the transforming growth factor beta receptor signaling pathway, bone morphogenetic protein (BMP) signaling pathway, MAPK signaling pathway, and apoptosis. Among them, TGF−β signaling has also been widely studied, and plays multiple roles in the regulation of apoptosis, inflammation, pancreatic regeneration, and repair in response to SAP (Gittes, 2016; Zhou et al., 2019). It was reported that the BMP signaling pathway was upregulated in the pancreases of AP mice and promotes the development of AP (Cao et al., 2013). Furthermore, as is well known, MAPK is one of the primary signaling pathways that triggers pro-inflammatory cytokines and oxidative stress and amplifies the inflammatory cascade in the progression of SAP (Pereda et al., 2006). These reports support the hypothesis that dysregulated circRNAs play important roles in the pathogenesis of SAP.

As is known, circRNAs can act as miRNA sponges to regulate the levels of miRNAs and inhibit their target genes through the ceRNA network. In our study, an interaction network between 18 circRNAs and miRNA was constructed in SAP, and it offered a large amount of important information that was used to research circRNAs and their regulated miRNAs. Among the ceRNA network, we mainly focused on CircZFP644 and its targeted miR-21-3p. The miR-21 family is known to be overexpressed in human specimens and experimental models in SAP, and plays an important role in the development and progression of the disease. It has been reported that miR-21-3p is overexpressed in the pathological state of acinar cells that have been overdosed with caerulein or sodium taurocholate rather than caerulein in physiological doses (Dixit et al., 2016). Additionally, the overexpression of miR-21-3p in acinar cells inhibited apoptosis (Wang et al., 2018), which nullifies the protective effects of apoptosis for preventing leakage of necrotic substances and limiting inflammation (Najenson et al., 2018), and also promotes necroptosis of pancreatic acinar cells (Ma et al., 2015), thereby aggravating the injury of pancreatic tissues. Additionally, it was confirmed by several studies that deficiency of miR-21 in mice contributed to amelioration of pancreatic injury during AP (Ma et al., 2015; Li et al., 2018). However, the upstream regulatory factors of miR-21 involved in SAP are not known yet.

In the present study, we discovered that there was a tendency for negative correlation and 3 binding sites between CircZFP644 and miR-21-3p. This suggests that the downregulated CircZFP644 may sponge miR-21-3p in SAP. Overall, our study demonstrates the potential role of CircZFP644 to modulate miR-21-3p-mediated regulation and thus SAP pathophysiology. Further study should be carried out to investigate the precise mechanism and specific function of CircZFP664 in SAP.

There are some limitations in our study. Due to the well-established SAP model, our results should be considered as an experimental study, and the results should be investigated in SAP patients. Additionally, our study mainly characterized one circRNA, CircZFP664, and predicted its role in the pathophysiology of SAP. Further studies should be carried out to investigate the precise mechanism of CircZFP664 in SAP. Additionally, we will explore the role of other circRNAs found in our study with *in vivo* and *in vitro* experiments.

## Conclusion

Our study reveals for the first time the circRNA expression profiles of pancreatic tissues and characterized a differentially expressed circRNA derived from the ZFP664 gene in SAP (Figure 9). We found that the CircZFP664 gene may be involved in the pathogenesis of SAP by sponging miR-21-3p. Our findings provide novel insights into understanding the development and progression of SAP, and delineate potential therapeutic targets of clinical interest.

## Data Availability Statement

The datasets generated for this study can be found in the Gene Expression Omnibus: GSE139173.

## Ethics Statement

The animal study was reviewed and approved by the Institutional Animal Care and Use Committee at the General Hospital of Western Theater Command.

## Author Contributions

HS, YY, and JR conceived and designed the study. QH, JW, XY, WJ, and YW performed experiments and analyzed data. YY and JR wrote the main manuscript text and prepared all of the tables and figures. LT and HS critically reviewed and proofread the manuscript. All authors reviewed the manuscript before submission and approved the final version of the manuscript.

## Conflict of Interest

The authors declare that the research was conducted in the absence of any commercial or financial relationships that could be construed as a potential conflict of interest.

## References

[B1] Ashburner M. Ball C. A. Blake J. A. Botstein D. Butler H. Cherry J. M. (2000). Gene ontology: tool for the unification of biology. The gene ontology consortium. *Nat. Genet.* 25 25–29. 10.1038/75556 10802651 PMC3037419

[B2] Beermann J. Piccoli M. T. Viereck J. Thum T. (2016). Non-coding RNAs in development and disease: background, mechanisms, and therapeutic approaches. *Physiol. Rev.* 96 1297–1325. 10.1152/physrev.00041.2015 27535639

[B3] Betel D. Wilson M. Gabow A. Marks D. S. Sander C. (2008). The microRNA.org resource: targets and expression. *Nucleic Acids Res.* 36 D149–D153. 10.1093/nar/gkm995 18158296 PMC2238905

[B4] Cao Y. Yang W. Tyler M. A. Gao X. Duan C. Kim S. O. (2013). Noggin attenuates cerulein-induced acute pancreatitis and impaired autophagy. *Pancreas* 42 301–307. 10.1097/MPA.0b013e31825b9f2c 22850625 PMC3894737

[B5] Chen S. Zhao Y. (2018). Circular RNAs: characteristics, function, and role in human cancer. *Histol. Histopathol.* 33 887–893. 10.14670/hh-11-969 29393503

[B6] Dixit A. K. Sarver A. E. Yuan Z. George J. Barlass U. Cheema H. (2016). Comprehensive analysis of microRNA signature of mouse pancreatic acini: overexpression of miR-21-3p in acute pancreatitis. *Am. J. Physiol. Gastrointest. Liver Physiol.* 311 G974–G980. 10.1152/ajpgi.00191.2016 27686613 PMC5130546

[B7] Ewing B. Hillier L. Wendl M. C. Green P. (1998). Base-calling of automated sequencer traces using phred. I. Accuracy assessment. *Genome Res.* 8 175–185. 10.1101/gr.8.3.175 9521921

[B8] Forsmark C. E. Swaroop Vege S. Wilcox C. M. (2016). Acute pancreatitis. *N. Engl. J. Med.* 375 1972–1981. 10.1056/NEJMra1505202 27959604 PMC13220086

[B9] Gao Y. Wang J. Zhao F. (2015). CIRI: an efficient and unbiased algorithm for de novo circular RNA identification. *Genome Biol.* 16:4. 10.1186/s13059-014-0571-3 25583365 PMC4316645

[B10] Gittes G. K. (2016). Multiple roles for TGFbeta receptor type II in regulating the pancreatic response in acute pancreatitis. *J. Pathol.* 238 603–605. 10.1002/path.4676 26608971

[B11] Han B. Chao J. Yao H. (2018). Circular RNA and its mechanisms in disease: from the bench to the clinic. *Pharmacol. Ther.* 187 31–44. 10.1016/j.pharmthera.2018.01.010 29406246

[B12] Kanehisa M. Furumichi M. Tanabe M. Sato Y. Morishima K. (2017). KEGG: new perspectives on genomes, pathways, diseases and drugs. *Nucleic Acids Res.* 45 D353–D361. 10.1093/nar/gkw1092 27899662 PMC5210567

[B13] Kechin A. Boyarskikh U. Kel A. Filipenko M. (2017). cutPrimers: a new tool for accurate cutting of primers from reads of targeted next generation sequencing. *J. Comput. Biol.* 24 1138–1143. 10.1089/cmb.2017.0096 28715235

[B14] Kong X. Y. Du Y. Q. Li L. Liu J. Q. Wang G. K. Zhu J. Q. (2010). Plasma miR-216a as a potential marker of pancreatic injury in a rat model of acute pancreatitis. *World J. Gastroenterol.* 16 4599–4604. 20857533 10.3748/wjg.v16.i36.4599 PMC2945494

[B15] Li X. Lin Z. Wang L. Liu Q. Cao Z. Huang Z. (2018a). RNA-Seq analyses of the role of miR-21 in acute pancreatitis. *Cell Physiol. Biochem.* 51 2198–2211. 10.1159/000495866 30537729

[B16] Li X. Yang L. Chen L. L. (2018b). The biogenesis, functions, and challenges of circular RNAs. *Mol. Cell* 71 428–442. 10.1016/j.molcel.2018.06.034 30057200

[B17] Liu R. H. Wen Y. Sun H. Y. Liu C. Y. Zhang Y. F. Yang Y. (2018). Abdominal paracentesis drainage ameliorates severe acute pancreatitis in rats by regulating the polarization of peritoneal macrophages. *World J. Gastroenterol.* 24 5131–5143. 10.3748/wjg.v24.i45.5131 30568390 PMC6288649

[B18] Ma X. Conklin D. J. Li F. Dai Z. Hua X. Li Y. (2015). The oncogenic microRNA miR-21 promotes regulated necrosis in mice. *Nat. Commun.* 6:7151. 10.1038/ncomms8151 25990308 PMC4440243

[B19] Najenson A. C. Courreges A. P. Perazzo J. C. Rubio M. F. Vatta M. S. Bianciotti L. G. (2018). Atrial natriuretic peptide reduces inflammation and enhances apoptosis in rat acute pancreatitis. *Acta Physiol.* 222:e12992. 10.1111/apha.12992 29117461

[B20] Peery A. F. Crockett S. D. Murphy C. C. Lund J. L. Dellon E. S. Williams J. L. (2019). Burden and cost of gastrointestinal, liver, and pancreatic diseases in the united states: update 2018. *Gastroenterology* 156 254–272.e11. 10.1053/j.gastro.2018.08.063 30315778 PMC6689327

[B21] Pereda J. Sabater L. Aparisi L. Escobar J. Sandoval J. Vina J. (2006). Interaction between cytokines and oxidative stress in acute pancreatitis. *Curr. Med. Chem.* 13 2775–2787. 10.2174/092986706778522011 17073628

[B22] Schmidt J. Rattner D. W. Lewandrowski K. Compton C. C. Mandavilli U. Knoefel W. T. (1992). A better model of acute pancreatitis for evaluating therapy. *Ann. Surg.* 215 44–56. 10.1097/00000658-199201000-00007 1731649 PMC1242369

[B23] Song Z. Huang Y. Liu C. Lu M. Li Z. Sun B. (2018). miR-352 participates in the regulation of trypsinogen activation in pancreatic acinar cells by influencing the function of autophagic lysosomes. *Oncotarget* 9 10868–10879. 10.18632/oncotarget.24220 29541382 PMC5834275

[B24] Stoll L. Sobel J. Rodriguez-Trejo A. Guay C. Lee K. Veno M. T. (2018). Circular RNAs as novel regulators of beta-cell functions in normal and disease conditions. *Mol. Metab.* 9 69–83. 10.1016/j.molmet.2018.01.010 29396373 PMC5870096

[B25] Wang T. Jiang L. Wei X. Liu B. Zhao J. Xie P. (2018). MiR-21-3p aggravates injury in rats with acute hemorrhagic necrotizing pancreatitis by activating TRP signaling pathway. *Biomed. Pharmacother.* 107 1744–1753. 10.1016/j.biopha.2018.08.164 30257393

[B26] Xiang H. Tao X. F. Xia S. L. Qu J. L. Song H. Y. Liu J. J. (2017). Targeting MicroRNA Function in acute pancreatitis. *Front. Physiol.* 8:726. 10.3389/fphys.2017.00726 28983256 PMC5613139

[B27] Xu H. Guo S. Li W. Yu P. (2015). The circular RNA Cdr1as, via miR-7 and its targets, regulates insulin transcription and secretion in islet cells. *Sci. Rep.* 5:12453. 10.1038/srep12453 26211738 PMC4515639

[B28] Xu T. Wu J. Han P. Zhao Z. Song X. (2017). Circular RNA expression profiles and features in human tissues: a study using RNA-seq data. *BMC Genom.* 18 (Suppl. 6):680. 10.1186/s12864-017-4029-3 28984197 PMC5629547

[B29] Yang Y. Huang Q. Luo C. Wen Y. Liu R. Sun H. (2019). MicroRNAs in acute pancreatitis: from pathogenesis to novel diagnosis and therapy. *J. Cell Physiol.* 235 1948–1961. 10.1002/jcp.29212 31552677

[B30] Zhang F. Zhang R. Y. Zhang X. Y. Wu Y. N. Li X. Y. Zhang S. (2018). Comprehensive analysis of circRNA expression pattern and circRNA-miRNA-mRNA network in the pathogenesis of atnerosclerosis in rabbits. *Aging US* 10 2266–2283. 10.18632/aging.101541 30187887 PMC6188486

[B31] Zhang J. Ning X. Cui W. Bi M. Zhang D. Zhang J. (2015). Transforming growth factor (TGF)-beta-induced microRNA-216a promotes acute pancreatitis via Akt and TGF-beta pathway in mice. *Dig. Dis. Sci.* 60 127–135. 10.1007/s10620-014-3261-9 25501921

[B32] Zhang Q. Wang J. Y. Zhou S. Y. Yang S. J. Zhong S. L. (2019). Circular RNA expression in pancreatic ductal adenocarcinoma. *Oncol. Lett.* 18 2923–2930. 10.3892/ol.2019.10624 31452773 PMC6676441

[B33] Zhong Y. X. Du Y. J. Yang X. Mo Y. Z. Fan C. M. Xiong F. (2018). Circular RNAs function as ceRNAs to regulate and control human cancer progression. *Mol. Cancer* 17:79. 10.1186/s12943-018-0827-8 29626935 PMC5889847

[B34] Zhou Q. Xia S. Guo F. Hu F. Wang Z. Ni Y. (2019). Transforming growth factor-beta in pancreatic diseases: mechanisms and therapeutic potential. *Pharmacol. Res.* 142 58–69. 10.1016/j.phrs.2019.01.038 30682425

[B35] Zhu H. Huang L. Zhu S. Li X. Li Z. Yu C. (2016). Regulation of autophagy by systemic admission of microRNA-141 to target HMGB1 in l-arginine-induced acute pancreatitis in vivo. *Pancreatology* 16 337–346. 10.1016/j.pan.2016.03.004 27017485

