# Supplementary material for: CircRNA Expression Profiles and the Potential Role of CircZFP644 in Mice With Severe Acute Pancreatitis via Sponging miR-21-3p
Source: Front Genet. 2020 Mar 12;11:206. doi: 10.3389/fgene.2020.00206 (PMC7081725; doi:10.3389/fgene.2020.00206)
Supplement: Supplementary file 1 [file Table_1.DOCX]

Supplementary Material

**Supplementary Table 1.** The circular RNAs with differentially expression in pancreatic tissue of SAP mice compared with control.

|  | Regulation | P-value | Fold Change | circBaseID | GeneName |
| --- | --- | --- | --- | --- | --- |
| chr17:39845973-39846348+ | up | 0.043467685 | 12.0452696 |  | Rn45s |
| chr7:131041069-131082668+ | up | 0.000449443 | 11.3350327 |  | Dmbt1 |
| chr5:137152228-137152461- | down | 3.21971E-05 | 153.8550399 |  |  |
| chr5:137152216-137152449- | down | 0.000136452 | 151.0785443 |  |  |
| chr3:113256960-113258035- | down | 0.000291701 | 60.3430441 |  | Amy2a5 |
| chr5:137152216-137152431- | down | 9.24975E-05 | 50.8952945 |  |  |
| chr5:137152231-137152446- | down | 9.24975E-05 | 50.8952945 |  |  |
| chr5:137152198-137152431- | down | 3.01171E-05 | 43.5148647 |  |  |
| chr16:13697333-13699075+ | down | 0.00031471 | 38.6370225 | mmu_circ_0000663 | Bfar |
| chr5:106634831-106638635- | down | 9.45053E-05 | 36.7018583 | mmu_circ_0001382 | Zfp644 |
| chr12:3617606-3648389+ | down | 0.006199775 | 31.1454623 | mmu_circ_0000349 | Dtnb |
| chr8:22098731-22098929+ | down | 0.000253954 | 30.171522 |  | Nek5 |
| chr10:50748806-50754122+ | down | 0.000333129 | 29.1302842 |  | Ascc3 |
| chr6:38818230-38819313- | down | 0.031033123 | 29.1302842 | mmu_circ_0001468 | Hipk2 |
| chr10:12436281-12455564- | down | 0.004942385 | 26.4666197 | mmu_circ_0000144 | Utrn |
| chr6:41522817-41524108+ | down | 0.00216531 | 25.7962885 |  | Prss2 |
| chr6:38440455-38470222+ | down | 0.001949537 | 25.4476472 |  | Ubn2 |
| chr9:108533587-108534618+ | down | 0.000667509 | 23.1207219 | mmu_circ_0001843 | Qrich1 |
| chr4:137404385-137423389- | down | 0.00233014 | 20.197811 |  | Cela3b |
| chr10:18092845-18094908+ | down | 0.007415905 | 19.0068679 | mmu_circ_0000149 | Reps1 |
| chr8:111686551-111687162- | down | 0.002195714 | 19.0068679 |  | Ctrb1 |
| chr10:75274017-75279961+ | down | 0.002171703 | 17.6444131 | mmu_circ_0000180 | Specc1l |
| chr17:39846591-39846761+ | down | 0.000740594 | 17.6444131 |  | Rn45s |
| chr6:41311270-41312349- | down | 0.000233856 | 17.6444131 |  | TCRB |
| chr6:41313401-41376859- | down | 0.000233856 | 17.6444131 |  | TCRB |
| chr7:131050010-131082586+ | down | 0.001561061 | 17.6444131 |  | Dmbt1 |
| chr7:68036188-68069079+ | down | 0.002774072 | 16.0310132 |  | Igf1r |
| chr1:52708164-52709755- | down | 0.016261817 | 14.8818744 | mmu_circ_0000037 | Mfsd6 |
| chr11:117773099-117773362- | down | 0.002114706 | 14.00438 |  | Tmc6 |
| chr1:128193460-128221703+ | down | 0.004755758 | 12.7238236 | mmu_circ_0008145 | R3hdm1 |
| chr11:22169035-22173006- | down | 0.000979246 | 12.7238236 |  | Ehbp1 |
| chr3:133137266-133149650- | down | 0.004755758 | 12.7238236 |  | Arhgef38 |
| chr7:97660813-97664766+ | down | 0.000979246 | 12.7238236 | mmu_circ_0001598 | Rsf1 |
| chr8:79371784-79372364- | down | 0.042287955 | 12.2847166 | mmu_circ_0001699 | Smad1 |
| chr9:22643745-22659145+ | down | 0.021859433 | 11.8117515 |  | Bbs9 |
| chr5:106618071-106666845- | down | 0.000308639 | 11.1152838 | mmu_circ_0001380 | Zfp644 |
| chr7:131066592-131082663+ | down | 0.000308639 | 11.1152838 |  | Dmbt1 |
| chr7:141638772-141639797- | down | 0.003844474 | 11.1152838 |  | Muc6 |
| chr8:111686549-111686730- | down | 0.002072486 | 11.1152838 |  | Ctrb1 |
| chr9:65794622-65795495- | down | 0.002072486 | 11.1152838 | mmu_circ_0001797 | Zfp609 |
| chrY:90793278-90793680+ | down | 0.037895006 | 10.3613313 |  | Erdr1 |
| chr5:23540375-23565265- | down | 0.03882421 | 10.3185154 | mmu_circ_0001325 | Srpk2 |
| chrY:90793277-90793679+ | down | 0.042986607 | 9.5287091 |  | Erdr1 |
| chr8:111686622-111687228- | down | 0.048968994 | 9.3749934 |  | Ctrb1 |
| chr10:125294595-125302081- | down | 0.000685324 | 8.8222066 |  | Slc16a7 |
| chr3:106531137-106539603- | down | 0.000685324 | 8.8222066 | mmu_circ_0001154 | Cept1 |
| chr4:145154569-145156782- | down | 0.000685324 | 8.8222066 |  | Vps13d |
| chr6:41304524-41396190+ | down | 0.000685324 | 8.8222066 |  | TCR-beta chain |
| chr6:41305048-41357441+ | down | 0.000685324 | 8.8222066 |  | TCR-beta chain |
| chr7:131050208-131066712+ | down | 0.000685324 | 8.8222066 |  | Dmbt1 |
| chr7:131058262-131082745+ | down | 0.000685324 | 8.8222066 |  | Dmbt1 |
| chr7:131072851-131079545+ | down | 0.000685324 | 8.8222066 |  | Dmbt1 |
| chr9:22643745-22679064+ | down | 0.000685324 | 8.8222066 | mmu_circ_0001755 | Bbs9 |
| chr9:45395110-45395283+ | down | 0.000685324 | 8.8222066 |  | Fxyd6 |
| chr17:6137211-6139156+ | down | 0.023368919 | 3.9681769 | mmu_circ_0000723 | Tulp4 |
| chr11:20725685-20727639- | down | 0.031975673 | 2.0197811 | mmu_circ_0000242 | Aftph |
